# Incidence of common opportunistic infections among HIV-infected children on ART at Debre Markos referral hospital, Northwest Ethiopia: a retrospective cohort study

**DOI:** 10.1186/s12879-020-4772-y

**Published:** 2020-01-16

**Authors:** Mamaru Wubale Melkamu, Mulugeta Tesfa Gebeyehu, Abebe Dilie Afenigus, Yitbarek Tenaw Hibstie, Belisty Temesgen, Pammla Petrucka, Animut Alebel

**Affiliations:** 1 Debre Markos Referral Hospital, P.O. Box 269, Debre Markos, Ethiopia; 2 College of Health Science, Debre Markos University, Debre Markos, Ethiopia; 3 College of Nursing, University of Saskatchewan, Saskatoon, Canada; 4 School of Life Sciences and Bioengineering, Nelson Mandela African Institute of Science and Technology, Arusha, Tanzania; 5 Faculty of Health, University of Technology Sydney, Sydney, Australia

**Keywords:** Anti-retroviral therapy, Children, HIV, Opportunistic infections, Ethiopia

## Abstract

**Background:**

Opportunistic infections (OIs) are the leading cause of morbidity and mortality among children living with human immunodeficiency virus (HIV). For better treatments and interventions, current and up-to-date information concerning occurrence of opportunistic infections in HIV-infected children is crucial. However, studies regarding the incidence of common opportunistic infections in HIV-infected children in Ethiopia are very limited. Hence, this study aimed to determine the incidence of opportunistic infections among HIV-infected children on antiretroviral therapy (ART) at Debre Markos Referral Hospital.

**Methods:**

A facility-based retrospective cohort study was undertaken at Debre Markos Referral Hospital for the period of January 1, 2005 to March 31, 2019. A total of 408 HIV-infected children receiving ART were included. Data from HIV-infected children charts were extracted using a data extraction form adapted from ART entry and follow-up forms. Data were entered using Epi-data™ Version 3.1 and analyzed using Stata™ Version 14. The Kaplan Meier survival curve was used to estimate the opportunistic infections free survival time. Both bi-variable and multivariable Cox proportional hazard models were fitted to identify the predictors of opportunistic infections.

**Results:**

This study included the records of 408 HIV-infected children-initiated ART between the periods of January 1, 2005 to March 31, 2019. The overall incidence rate of opportunistic infections during the follow-up time was 9.7 (95% CI: 8.13, 11.48) per 100 child-years of observation. Tuberculosis at 29.8% was the most commonly encountered OI at follow-up. Children presenting with advanced disease stage (III and IV) (AHR: 1.8, 95% CI: 1.2, 2.7), having “fair” or “poor” ART adherence (AHR: 2.6, 95% CI: 1.8, 3.8), not taking OI prophylaxis (AHR:1.6, 95% CI: 1.1, 2.4), and CD4 count or % below the threshold (AHR:1.7, 95% CI: 1.1, 2.6) were at a higher risk of developing opportunistic infections.

**Conclusions:**

In this study, the incidence rate of opportunistic infections among HIV-infected children remained high. Concerning predictors, such as advanced disease stage (III and IV), CD4 count or % below the threshold, “fair” or “poor” ART adherence, and not taking past OI prophylaxis were found to be significantly associated with OIs.

## Background

The epidemic of Human Immunodeficiency Virus (HIV) remains a serious public health concern [1]. In 2018, an estimated 37.9 million people globally were living with HIV, including 1.7 million children (aged < 15 years) [2]. Sub-Saharan Africa (SSA) is the most affected region [3] with Ethiopia ranking within the top 25 countries with the highest new HIV-infection rates [4]. Opportunistic infections (OIs) are infections, which occur more frequently and severely among individuals with weakened immune systems, including People Living with Human Immunodeficiency Virus (PLHIV) [5]. All HIV-infected people are susceptible to develop a wide range of OIs [6], but prevalence and incidence of HIV-associated OIs vary widely [7, 8]. Most reports on the magnitude of OIs in HIV-infected children are from North America and Europe; while, in SSA, the true burden of OIs among HIV-children remains poorly documented [9] .

OIs are the leading causes of morbidity and mortality among HIV-infected children contributing to 94.1% of HIV-related deaths [10–13]. Unless OIs are treated as early as possible, they markedly affecting the treatment outcomes of PLHIV leading to poor quality of life, hastening disease progression, increasing medical costs, potentiating the risk of treatment failure, and impairing patient’s response to antiretroviral therapy (ART) drugs [14, 15]. In low and middle income countries, the most frequently occurring OIs are tuberculosis (TB), oral candidiasis, varicella zoster, pneumocystis pneumonia, bacterial pneumonia, herpes zoster, and dermatophyte infections [16, 17].

The World Health Organization (WHO) recommends a range of medical interventions to reduce the occurrence of OIs among PLHIV. These interventions include reduction of exposure, chemoprophylaxis (primary/secondary), immunization, and early initiation of ART [18]. The use of highly active antiretroviral therapy (HAART) has been effective in reducing OIs significantly among children and adolescents infected with HIV [19]. In Ethiopia, the Ministry of Health (MOH) has been implementing different interventions to improve the survival of HIV infected individuals which is reflected, in part, in the increased ART coverage from 5% in 2010 to 9.5% in 2013 [18, 20].

Despite the dramatic decline in the incidence OIs after the introduction HAART, they remain a major cause of morbidity and mortality among these vulnerable population [21]. Most HIV-associated OIs and other co-morbidities are easily preventable and treatable with safe and cost-effective interventions. However, for better interventions, information regarding the patterns and occurrences of OIs is essential for all age groups (especially vulnerable populations such as children and pregnant women), but are often absent for low and middle income countries, including Ethiopia. Therefore, this study was undertaken to determine the incidence of common OIs among HIV-infected children at Debre Markos Hospital. Results obtained from this study will potentially help health professionals to develop a new strategy for the prevention and management of OIs and inform a national program of research inclusive of interventional studies.

## Methods

### Study design, setting, and period

A facility-based retrospective cohort study was conducted at Debre Markos Referral Hospital. Debre Markos town is located 300 km far from Addis Ababa, the capital city of Ethiopia and 265 km far from Bahir Dar, the capital city of Amhara Region. According to the 2007 national census, Debre Markos has a total population of 62,497, of whom 29,921 were men and 32,576 were women [22]. Debre Markos Referral Hospital is the only referral hospital in East Gojjam Zone providing services for more than 3.5 million peoples in the zone and neighboring zones. The hospital has been providing HIV-care and ART follow-up services since 2005. Currently, the ART clinic of this hospital has one medical doctor, five nurses, three data clerks, one porter, one cleaner, five case managers, and six adherence supporters. The hospital uses standardized ART monitoring and evaluation tools adapted from the Ethiopian national comprehensive HIV care and treatment guidelines [18]. To date, a total of 466 HIV-infected children commenced ART at this site of which, approximately 350 HIV-infected children are receiving active ART follow-up at the time of this study.

### Study participants

The source population for this study were all HIV-infected children (aged < 15 years) ever initiated on ART at Debre Markos Referral Hospital, with the study population including those HIV-infected children started on ART between January 1, 2005 to March 31, 2019. HIV-infected children who had incomplete baseline information (CD4 count, hemoglobin level, WHO clinical stage, weight and height) and/or who had OIs at the time of ART initiation were excluded from the study.

### Sampling procedures

The records of all HIV-infected children ever started on ART (466) at Debre Markos Referral Hospital were recruited. After excluding incomplete records, records of 408 HIV-infected children met the criteria and were included in the study. Data were extracted from the charts of 408 HIV-infected children on ART.

### Variables of the study

The dependent variable for this study was the occurrence of any type of opportunistic infections during follow-up. The independent variables included: Socio-demographic characteristics (i.e., age, sex, residence, religion, marital status of care giver, relationship of care giver, current status of parents, occupation of the caregiver, and family size); Clinical and laboratory predictors (i.e., WHO clinical stage, CD4 count, hemoglobin (Hgb) level, underweight, wasting, stunting, history of Prevention of Mother to Child Transmission (PMTCT), prior history of OIs, functional status, and developmental status); and ART and other medication-related predictors (i.e., past OI prophylaxis, type of baseline ART regimen, ART eligibility criteria, presence of regimen changed, level of ART adherence, ever taking Isoniazid Preventive Therapy (IPT), ART side effects, and ART treatment failure).

### Operational definition of variables

In this study, an event was considered when HIV-infected child developed any form of OIs after ART initiation during the follow-up period.

Censored was recorded when HIV-infected children dropped or transfer out (whether dead or alive) to other health institutions or are still on active ART follow-up, but did not develop any OIs by the end of the study.

ART adherence was classified as good, fair, or poor, according to the percentage of drug dosage calculated from the total monthly dose of ART drugs. Good was reported with compliance equal to or greater than 95% or ≤ 3 missed doses per month; fair reflected 85–94% compliance and between 4 and 8 missing doses per month); and poor reflected less than 85% compliance or ≥ 9 missed dose per month) [23].

Child developmental status was classified as appropriate (able to attain milestones for age), delayed (failure to attain milestones for age); and regression (loss of what has been attained for age) [18].

Children with weight/age Z-score < − 2 SD, height/age Z-score < − 2 SD, and weight/height Z-score < − 2 SD were considered to be underweight, stunted, and wasted respectively [24, 25].

OIs are infections, which occur more frequently and severely among individuals with weakened immune systems, including People Living with Human Immunodeficiency Virus (PLHIV) [5]. According to the Ethiopian ART guidelines, the common OIs include herpes zoster, bacterial pneumonia, pulmonary TB, extra-pulmonary TB, oral candidiasis, oesophageal candidiasis, mouth ulcer, diarrhoea, pneumocystis pneumonia, central nervous system toxoplasmosis, Cryptococcal meningitis, non-Hodgkins lymphoma, Kaposi’s sarcoma, cervical cancer, and others [18].

CD4 counts or percentage (%) below the threshold was considered if the child had CD4 cell counts < 1500/mm^3^ or 25% for age < 12 months, CD4 cell counts < 750/mm^3^ or < 20% for age 12–35 months, CD4 cell counts < 350/mm^3^ or < 15% for age 36–59 months, and CD4 cell counts < 200/mm^3^ or < 15% for age ≥ 60 months [26].

### Data collection procedures and quality assurance

The data extraction form was based on the federal Ministry of Health’s HIV-care/ART follow-up and intake forms, which are used in the ART clinics of Ethiopian hospitals. The data extraction form included the following variables: socio-demographic characteristics, ART and other medication, clinical, and laboratory-related information. Data were collected by three BSc prepared nurses working in the ART clinic of Debre Markos Referral Hospital. To assure data quality, a data extraction checklist was carefully adapted from a standardized ART intake and follow up forms, nurses currently work in the ART clinic and who took ART training were recruited as data collectors, a one-day training was given for both data collectors and supervisors, completeness of the recorded variables were double checked by taking some randomly selected cards, and the supervisor as well as principal investigators carefully monitored the entire data collection process. Subsequently, all relevant data were retrieved through reviewing of HIV-infected children’s cards. The occurrence of OIs during data extraction was ascertained by reviewing the health professionals’ reporting on patient charts. Any laboratory tests obtained at the time of ART initiation were considered as a baseline data. However, if laboratory tests were not done during ART initiation, any lab tests done within a month of ART initiation were considered as the baseline.

### Data analysis

Before entry, data were checked for its completeness and consistency. We used Epi-data™ Version 3.1 for data entry and STATA™ Version 14 for data analysis. Besides, Emergency Nutritional Assessment (ENA) and WHO AnthroPlus software were used to analyze the nutritional status (underweight [WAZ], stunting [HAZ] and wasting [WHZ]) of HIV-infected children. Descriptive statistics were summarized as percentage, mean, and median, and visualized using tables and charts. The OIs free survival time was estimated using the Kaplan-Meier survival curve. Besides, the OIs free survival time between different categorical explanatory variables were compared using generalized log rank test. A life table was constructed to estimate probabilities of OIs at different time intervals and cumulative survival probabilities. The common assumptions of Cox proportional hazard regression model were checked using Schoenfeld residuals test. In the bi-variable analysis, variables with *p*-values ≤0.25 were selected for the multivariable analysis [27, 28]. In the final model, variables with *p*-values < 0.05 were considered as statistically significant predictors of OIs.

## Results

### Socio-demographic characteristics of the cohort

A total of 466 HIV-infected children’s medical records were retrieved. Of these, 58 were excluded from the analysis due to incompleteness. The remaining 408 HIV-infected children on ART were included in this study. The median age of the study participants was 7 years (IQR = 4, 10). About 42.9% of the children were in the age group of 5 to 9 years. Slightly more than half (54.7%) of the children were male, and more than two thirds (72.1%) were from urban areas. More than half (53.7%) of the parents were both alive and the majority (85.3%) of children were living with their biological parents **(**Table 1**).**

**Table 1:** Sociodemographic characteristics of HIV-infected children on ART at Debre Markos Referral Hospital, Northwest Ethiopia, from January 1, 2005 to March 31, 2019 (*N* = 408)

Characteristics	Category	Frequency (N)	Percentage (%)
Age of the child (years)	*0–4 years*	122	29.9
	*5–9 years*	175	42.9
	*10–14 years*	111	27.2
Sex	Male	223	54.7
	Female	185	45.3
Residence	Urban	294	72.1
	Rural	114	27.9
Family size	1–3	184	45.1
	≥4	179	43.9
	Not recorded	45	11.0
Current status of parents	Both alive	219	53.7
	One or both deceased	189	46.3
Marital status of the caregiver	*Married*	172	42.0
	*Divorced/ widowed*	167	41.0
	*Not recorded*	69	17.0
Occupation of the caregiver	Employed	86	21.3
	Unemployed	203	50.3
	Merchant	65	16.1
	Not recorded	54	12.4
With whom the child lives	Biological	348	85.3
	Non-biological	60	14.7
Religion	Orthodox	348	85.3
	Muslim	21	5.1
	Others	39	9.5

### Clinical, laboratory and medication-related characteristics

About half (51.9%) of children were classified as WHO clinical stage I and II and almost two third (67.9%) of the children had CD4 counts or percentage (%) above the threshold. About 10.3% of participants had < 10 mg/dl hemoglobin level with a median of 12.3 (IQR = 11.3–13.3) mg/dl, with 49.3, 23.8, and 51.7% of participants being underweight, wasted and stunted respectively. More than half (60.8%) of children had taken past OI prophylaxis. Children exhibiting a good adherence level to ART and nevirapine baseline ART regimens were 78.7 and 66.7%, respectively and 56.1% had no history of PMTCT **(**Table 2**).**

**Table 2:** Baseline clinical, laboratory and medication-related characteristics of the HIV-infected children on ART at Debre-Markos Referral Hospital, Northwest Ethiopia, from January 1, 2005 to March 31, 2019 (*N* = 408)

Characteristics	Categories	Frequency (N)	Percentage (%)
History of PMTCT	Yes	56	13.7
	No	229	56.1
	Not recorded	123	30.2
History of OIs	*Yes*	215	52.7
	*No*	193	47.3
Weight for age	Normal	207	50.7
	Underweight	201	49.3
Weight for height	Normal	311	76.2
	Wasted	97	23.8
Height for age	Normal	197	48.3
	Stunted	211	51.7
WHO clinical stage	Stage I and II	212	52.0
	Stage III and IV	196	48.0
CD4 counts or %	Above the threshold	277	67.9
	Below the thresheold	131	32.1
Hgb level	*≥ 10 g/dl*	366	89.7
	*< 10 g/dl*	42	10.3
ART eligibility criteria	Test and treat	67	16.4
	CD4 or WHO staging	341	83.6
Type of regimen at baseline	Nevirapine base	272	66.7
	Efaveraze base	86	21.1
	Protease inhibitor base	50	12.3
Ever taking IPT	Yes	217	53.2
	No	191	46.8
ART adherence	Good	321	78.7
	Fair/ poor	87	21.3
Past OI prophylaxis	Yes	248	60.8
	No	160	39.2
ART side effect	Yes	19	4.7
	No	389	95.3
Regimen change	Yes	84	20.6
	No	324	79.4
Treatment failure	Yes	20	4.9
	No	388	95.1
Functional status	Working	153	53.3
	Ambulatory	113	39.4
	Bed ridden	21	7.3
Developmental status	Appropriate	73	60.3
	Delayed	41	33.9
	Regressed	7	5.8

*PMTCT* Prevention of Mother To Child Transmission, *OIs* Opportunistic Infections, *WHO* World Health Organization, *Hgb* Hemoglobin, *CD4* Cluster of differentiation 4, and *ART* Antiretroviral Therapy

### Incidence of opportunistic infections during follow-up

**Fig. 1:**
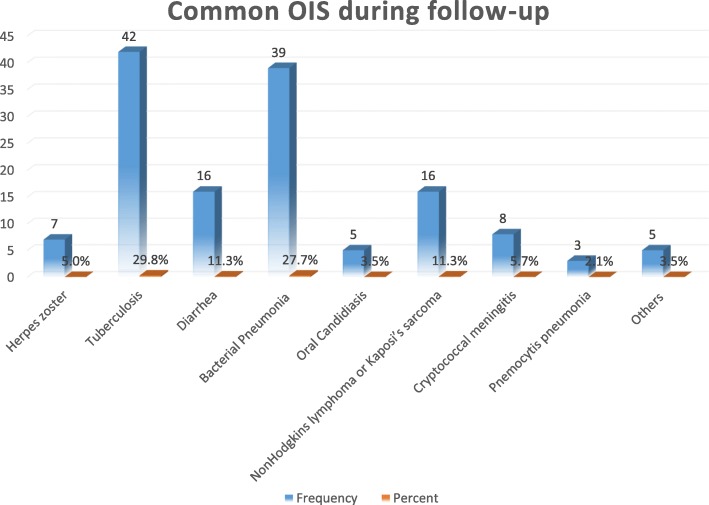
Common types of OIs during follow-up time among HIV-infected children at Debre Markos Referral Hospital from 2005 to 30th March, 2019

The study participants were followed for a minimum of 2 months and a maximum of 132 months. The total person months of the cohort was 16, 024 child-months of observation. During the follow-up time, almost one third (31.6%) of the study participants developed OIs. This study found that the incidence rate of OIs among HIV-infected children was 9.7 (95% CI: 8.1, 11.5) per 100 child-years of observation. From all types of OIs occurring during the follow-up time, TB (29.8%) was the most common, followed by bacterial pneumonia (27.7%), and non-Hodgkins lymphoma or Kaposi’s sarcoma (11.3%) (Fig. 1).

### Opportunistic infections free survival time of HIV-infected children on ART

**Fig. 2:**
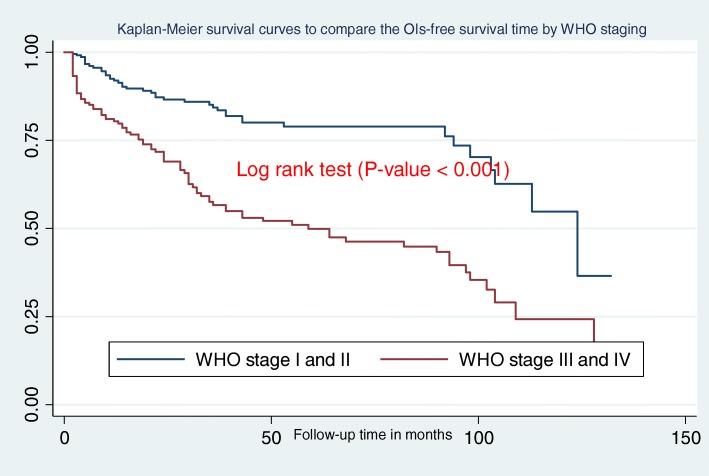
Kaplan-Meier survival curves to compare the OIs-free survival time of HIV-infected children on ART with different categories of WHO clinical stages at Debre-Markos Referral Hospital from 2005 to 30th March, 2019

**Fig. 3:**
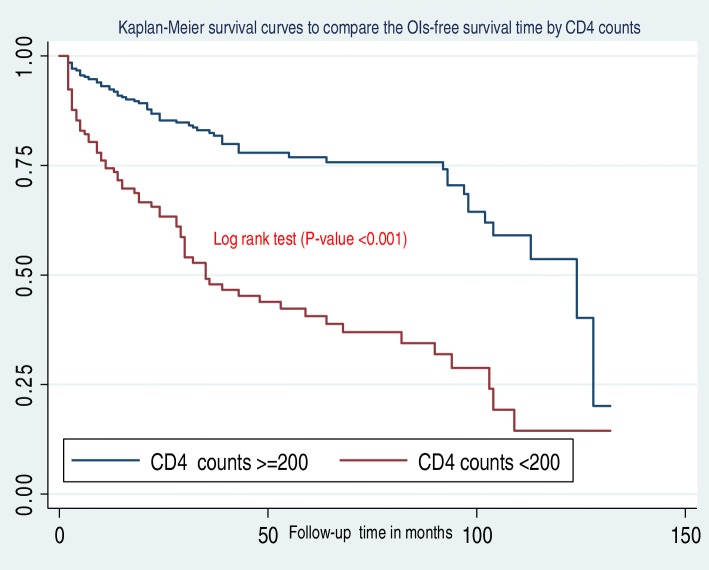
Kaplan-Meier survival curves to compare the OIs-free survival time of HIV-infected children on ART with different categories of CD4 counts or % at Debre-Markos Referral Hospital from 2005 to 30th March, 2019

**Fig. 4:**
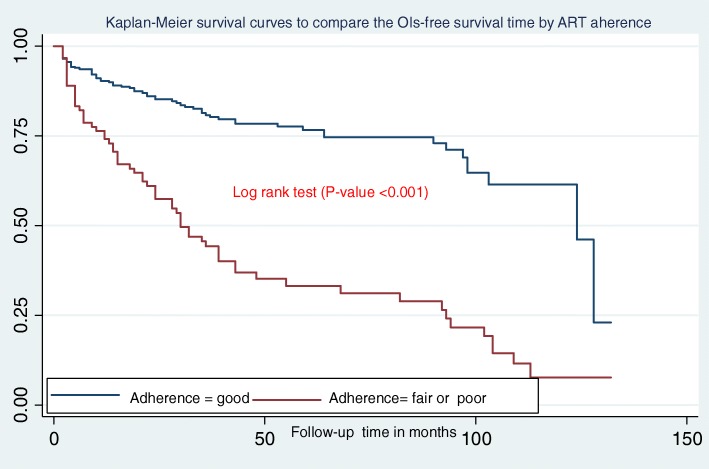
Kaplan-Meier survival curves to compare the OIs-free survival time of HIV-infected children on ART with different categories of ART drug adherence at Debre-Markos Referral Hospital from 2005 to 30th March, 2019

**Fig. 5:**
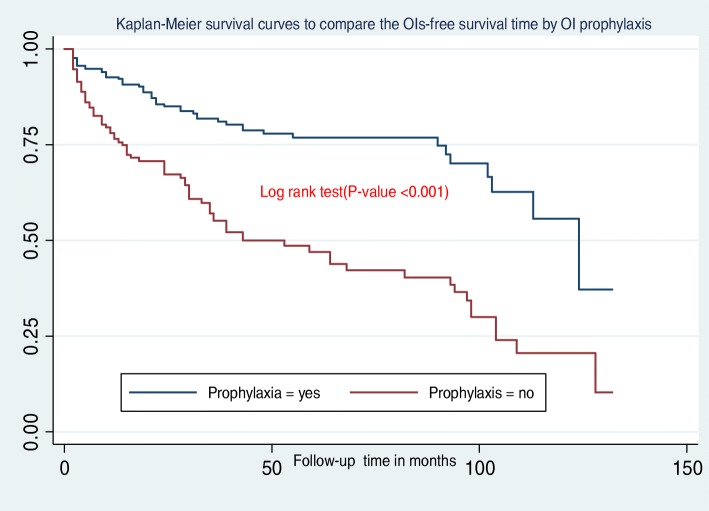
Kaplan-Meier survival curves to compare the OIs-free survival time of HIV-infected children on ART with different categories of OI prophylaxis at Debre-Markos Referral Hospital from 2005 to March 2019

In this study, the median OIs-free survival time was 103 months (IQR = 30,128)**.** Additionally, children presenting with WHO clinical stage III and IV during ART initiation had less OIs free survival time when compared to children presenting in WHO stages I and II **(**Fig. 2**)**. Figure **3** shows that the OIs free survival time of children presenting with severe immunodeficiency (CD4 count or %bellow the threshold) was lower than those children with mild immunodeficiency (CD4 count or % above the threshold). Moreover, children who had “fair or poor” ART drug adherence had less OIs free survival time as compared to those who had good ART drug adherence **(**Fig. 4**)**. Furthermore, children who did not take past OI prophylaxis had less OIs free survival time as compared to the past OI prophylaxis user cohort **(**Fig. 5**)**.

### Predictors of OIs among HIV-infected children on ART

Weight for age Z-scores, history of past OIs, Hgb levels, WHO clinical staging, CD4 counts or %, taking past OI prophylaxis, ever taking IPT, and ART drug adherence were variables for multivariable analysis Of these, WHO clinical staging, CD4 counts, ART drug adherence, and past OIs prophylaxis were found to be significant predictors of OIs. Children presenting with WHO clinical stage III and IV were nearly 2 times (AHR (adjusted hazard ratio): 1.8, 95% CI: 1.2, 2.7) more likely to develop OIs as compared to those children in WHO clinical stages I and II. This study also found that HIV-infected children on ART who had “fair” or “poor” ART adherence were 2 and half fold (AHR: 2.6, 95% CI: 1.8, 3.8) more likely to develop OIs as compared to those children who had “good” ART adherence. The risk of developing OIs among children with CD4 count or % below the threshold was 1.7 times (AHR: 1.7, 95% CI: 1.1, 2.6) higher as compared to those children with CD4 counts or % above the threshold. Lastly, the risk of developing OIs among children who did not previously take OI prophylaxis was 1.6 times (AHR: 1.6, 95% CI: 1.1, 2.4) higher to those who took past OI prophylaxis **(**Table 3**).**

**Table 3:** The bi-variable and multivariable Cox-regression analysis of the predictors of OIs among HIV-children on ART at Debre-Markos Referral Hospital, Northwest Ethiopia from January 1, 2005 to March 31, 2019 (*N* = 408)

Variables	Events	Censored	CHR (95% CI)	AHR (95% CI)
Age (years)				
0–4 years	36	86	1	1
5–9 years	63	112	1.5 (0.98, 2.3)	1.2 (0.8, 1.9)
10–14 years	30	81	1.7 (1.03, 2.9)	1.4 (0.8, 2.4)
Current status of parents				
Both alive	59	160	1	1
One or both dead	70	119	1.3 (0.9, 1.8)	1.0 (0.7, 1.5)
History of OIs				
No	45	148	1	1
Yes	84	131	1.8 (1.3, 2.6)	1.2 (0.8, 1.83)
Weight for age Z-score				
Normal weight	55	152	1	1
underweight	74	127	1.4 (1.0, 2.0)	1.1 (0.7, 1.6)
Height for age Z-score				
Normal	56	141	1	1
Stunted	73	138	1.3 (0.9, 1.9)	0.8 (0.5, 1.1)
WHO clinical stage				
Stage I and II	41	176	1	1
Stage III and IV	88	103	2.9 (2.0, 4.1)	1.8 (1.2, 2.7)^a^
CD4 counts or %				
Above the threshold	60	217	1	1
Below the thresheold	69	62	3.3 (2.3, 4.6)	1.7 (1.1, 2.6)^a^
Hgb level				
≥ 10 g/dl	109	257	1	1
< 10 g/dl	20	22	1.7 (1.1, 2.8)	1.0 (0.6, 1.7)
Ever taking IPT				
Yes	64	162	1	1
No	65	117	1.9 (1.3, 2.7)	1.2 (0.8, 1.7)
ART adherence				
Good	65	252	1	1
Fair/poor	64	27	3.7 (2.6, 5.2)	2.6 (1.8–3.8)^a^
Past OI prophylaxis				
Yes	51	204	1	1
No	78	75	2.8 (2.0, 4.0)	1.6 (1.1, 2.4)^a^

^a^*significant predictors*

## Discussion

This facility-based retrospective cohort study was undertaken to determine the incidence of common OIs among HIV-infected children on ART at Debre Markos Referral Hospital. Almost one third (31.6%) of the study participants developed OIs, yielding an incidence rate of OIs 9.7 (95% CI: 8.1, 11.5) per 100 child-years of observation. This finding is comparable with an Asian-based study reporting 10.5 per 100 person-years [21]. Similarly, our finding is much higher than studies from the United States of America (4.99 per 100 person-years) [29], Latin America (1.1 per 100 person-years) [30], and Brazil (2.63 per 100 person-years) [10].

Literature also showed that HIV-related OIs remain high in resource limited settings; especially with SSA being disproportionately affected [9]. Developed countries have advanced technologies for early diagnosis, prevention, and management of OIs. Additional explanations for the above noted discrepancies might be attributed to the lack of awareness among HIV-infected peoples living in developing countries to take ART medications and OIs prophylaxis continuously [31]. Poverty, overcrowding, and malnutrition are common problems in developing countries, which could contribute to the higher occurrence of OIs among HIV-infected people.

Among all types of OIs, TB is the most common (29.8%) during the follow-up time. This finding is consistent with a study conducted in India, which documented that TB is the most (34.6%) commonly diagnosed OI among HIV-infected individuals [32].However, studies reported from Latin America [16, 17, 29] revealed that bacterial pneumonia is the most commonly diagnosed OI [33, 34]. A study done in India illustrated that pulmonary TB was the most common opportunistic infection and accounted for 26% of all OI cases [35]. PLHIV are 20 to 37 times more likely to develop TB as compared to the general population [18]. Besides, Cryptococcal meningitis accounted for 5.7% of OIs among HIV-infected children on ART at Debre Markos Referral Hospital. This is also an important finding since *Cryptococcus neoformans* is not a common cause of meningitis in children.

In this study, children who started ART as WHO clinical stage III and IV were more likely to develop OIs as compared to those children who started ART as WHO stage I and II. This finding aligns with studies done in India [36, 37], and Asia [21]. OIs tend increase in number and severity in alignment with WHO clinical disease staging [36]. WHO clinical stages and OIs have a positive association [37], such that HIV-infected individuals with WHO clinical stage III and IV often exhibit severe immune deficiency [38]. The level of immunity determines the occurrence and type of OIs [18]. Therefore, as the WHO clinical stage becomes more advanced the occurrence and recurrence of OIs also increase. The most serious and life threatening OIs become more common among HIV-infected peoples with stage III and IV.

This study also found that ART drug adherence is an important predictor for OIs. Children who had “fair or poor” ART drug adherence were more likely to develop OIs as compared to those who had good ART drug adherence. A study conducted in Cameron found that HIV-infected patients who were not adhered to the ART therapy were more prone to develop OIs. This study also documented that OIs significantly increased the risk of non-adherence [39].

HIV-infected children with CD4 count or % below the threshold at the time of ART initiation were more likely to develop OIs as compared to those children with CD4 counts or % above the threshold). A 10 year retrospective study conducted in Uganda [40] documented that the occurrence of OIs in patients with lower CD4 cell counts at ART start was significantly high. Similar studies conducted in India [32, 36], Latin America [29, 30, 41], and Asia [21] showed that low CD4 counts during ART enrollment significantly increased the risk of developing OIs. A study from the United States of America revealed that children who had a CD4 percentage of less than 15% at ART initiation were at higher risk of OIs compared to those children who had an on initiation CD4 percentage at or greater than 25% [29]. CD4 cells are a type of white blood cells, which help to activate other white blood cells in the immune system. Therefore, any factors which reduce the number of CD4 cells will impair the immune system of HIV-infected children exposed to the development of OIs [42].

Lastly, this study found that the risk of developing OIs among HIV-infected children who took past OI prophylaxis was low as compared to those HIV-infected children who did not take past OI prophylaxis. Previous Ethiopian studies revealed that prophylaxis has a significant effect in reducing the occurrence of TB [43–45]. Another supportive finding was documented in Latin America [30]. Cotrimoxazole preventive therapy (CPT) is a feasible, cost-effective, and safe way intervention to reduce HIV/AIDS related morbidities and mortalities associated with OIs [45]. Furthermore, the Ethiopian ART guideline recommends early initiation of CPT among HIV-infected children benefited for the prevention of OIs like pneumocystis pneumonia, bacterial infections, toxoplasmosis gondii, diarrhea and other protozoal infections; IPT to address latent TB, and fluconazole for prevention of fungal and cryptococcal infections [18].

### Limitations of the study

The current study has some limitations, which must be considered before interpreting results. The data for this study was obtained from charts of HIV-infected children. Therefore, we were unable to get some variables including viral load, income, micronutrient deficiency, and immunization status of the children. At times, OIs might be underestimated due to excluded charts with incomplete data and unrecorded by health professions. Although the data are not always complete, the longitudinal data set may have corrected for some of these limitations.

## Conclusions

In this study, the incidence rate of opportunistic infections among HIV-infected children remained high. Concerning predictors such as advanced disease stage (III and IV), CD4 count or % below the threshold, “fair” or “poor” ART adherence, and not taking past OI prophylaxis were found to be significantly associated with OIs. Therefore, based on the above findings, we recommend consideration should be given to early screening and treatment of OIs. Besides, adherence support through phone calls and case managers could be strengthened. Furthermore, children presented with severe immunodeficiency and advanced disease stage during the ART initiation should be closely monitored and deeply investigated for the occurrence of OIs in each successive follow-up.

## Data Availability

Data will be available upon request of the corresponding author.

## Abbreviations

AIDS: Acquired Immune Deficiency Syndrome
ART: Antiretroviral Therapy
AZT- 3TC-EFV: Zidovudine plus Lamivudine plus Efavirenz
AZT-3TC-NVP: Zidovudine plus Lamivudine plus Nevirapine
CPT: Cotrimoxazole Preventive Therapy
HAART: Highly Active Antiretroviral Therapy
HAZ: Height for Age Z-score
Hgb: Hemoglobin
HIV: Human Immunodeficiency Virus
IPT: Isoniazid Preventive Therapy
OIs: Opportunistic Infections
PMTCT: Prevention of Mother to Child Transmission
SSA: Sub-Saharan Africa
TB: Tuberculosis
WAZ: Weight for Age Z-score
WHO: World Health Organization
WHZ: Weight for Height Z score
